# Supplementary material for: Identification of photoperiod-induced specific miRNAs in the adrenal glands of Sunite sheep (Ovis aries)
Source: Front Vet Sci. 2022 Jul 22;9:888207. doi: 10.3389/fvets.2022.888207 (PMC9354845; doi:10.3389/fvets.2022.888207)
Supplement: Supplementary file 1 [file Data_Sheet_1.ZIP › Supplementary file/miRDeep2 file.zip!/miRDeep2 file/Know_Structure/SP42A2/oar-mir-323a.pdf]

miRBase precursor	: oar-mir-323a
Total read count	: 3069
oar-miR-323a-5p read count	: 19
oar-miR-323a-3p read count	: 3049
remaining reads	: 1

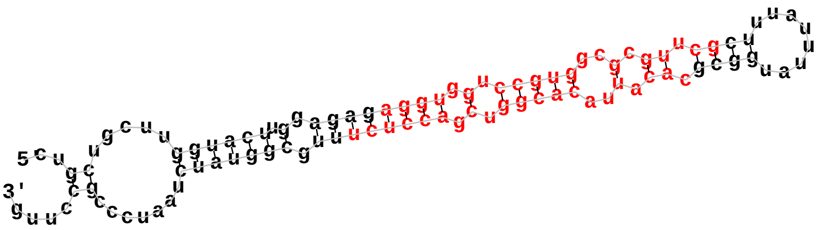

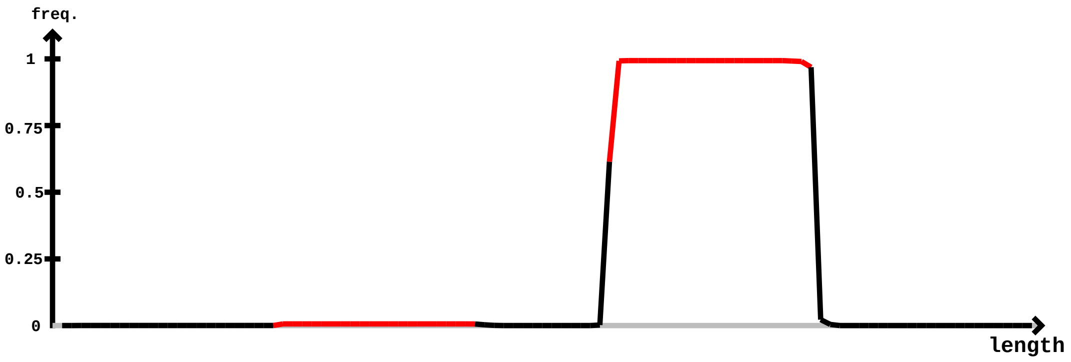

oar-miR-323a-5p

oar-miR-323a-3p

5'- cugcugcuugguacuuggagagagguggucc guggcgcguucgcuuuauuuauggcgcacauuacacggucgaccucuuugcgguaucuaaucccgccuug -3'	exp		
.........((((((.(.((((((((.(.(((((..(.((.((((........)))).)).)..))))).).)))))))).)))))))............	reads	mm	sample
.ugcugcuugguacuuggagag...	1	0	seq
......................agguggucc guggcgcguuc...	1	0	seq
......................agguggucc guggcgcguucg..	9	0	seq
......................agguggucc guggcgcguucgc...	7	0	seq
......................agguggucc guggcgcguucgcu..	2	0	seq
....cgcacauuacacggucgaccucu.......................	5	0	seq
....cgcacauuacacggucgaccucuu......................	1	0	seq
....gcacauuacacggucgacc..........................	3	0	seq
....gcacauuacacggucgaccu.........................	2	0	seq
....gcacauuacacggucgaccuc........................	37	0	seq
....gcacauuacacggucgaccucu.......................	1803	0	seq
....gcacauuacacggucgaccucuu......................	34	0	seq
....gcacauuacacggucgaccucuuu.....................	1	0	seq
....cacauuacacggucgacc..........................	2	0	seq
....cacauuacacggucgaccu.........................	3	0	seq
....cacauuacacggucgaccuc........................	29	0	seq
....cacauuacacggucgaccucu.......................	1094	0	seq
....cacauuacacggucgaccucuu......................	20	0	seq
....cacauuacacggucgaccucuuu.....................	12	0	seq
....acauuacacggucgaccucu.......................	3	0	seq
